# Metabolomic and Transcriptomic Analysis Reveal the Role of Metabolites and Genes in Modulating Flower Color of *Paphiopedilum micranthum*

**DOI:** 10.3390/plants12102058

**Published:** 2023-05-22

**Authors:** Xinyan Deng, Chao Hu, Chengzhi Xie, Aixian Lu, Yibo Luo, Tao Peng, Weichang Huang

**Affiliations:** 1 School of Life Sciences, Guizhou Normal University, Guiyang 550025, China; dengxinyan339@163.com; 2 Eastern China Conservation Centre for Wild Endangered Plant Resources, Shanghai Chenshan Botanical Garden, Shanghai 201602, China; hc320320@163.com (C.H.); bjfuxcz@163.com (C.X.); aixianlu0822@163.com (A.L.); 3 College of Forestry, Hainan University, Haikou 570228, China; 4 State Key Laboratory of Systematic and Evolutionary Botany, Institute of Botany, Chinese Academy of Sciences, Beijing 100000, China; 5 China National Botanical Garden, Beijing 100000, China

**Keywords:** floral color, floral signal, anthocyanin, carotenoid, food deception, orchids

## Abstract

Food-deceptive flowers primarily use visual signals (such as color) to mimic model plants and deceive insects into achieving pollination. *Paphiopedilum micranthum* is a food-deceptive orchid that has a pink labellum and two purple petals with a yellow base and has been proven to be pollinated by bumblebees. However, the chemical and molecular bases of the floral color are not well understood. We conducted targeted metabolite profiling and transcriptomic analysis to determine the color signal and its genetic basis in *P. micranthum*. We found that both anthocyanins and carotenoids contribute significantly to the formation of floral color that determines the color signal. Higher concentrations of anthocyanins (cyanidin and peonidin) and carotenoids (primarily lutein and zeaxanthin) were detected in the petal compared to the labellum. The upregulation of structural genes of *CHS*, *F3*′*H*, *DFR* and *ANS* on the anthocyanin biosynthesis pathway in petals was identified, as well as three genes of *LCYE*, *BCH*, and *CCD4* on the carotenoid biosynthesis pathway. Furthermore, we discovered that three *R2R3-MYBs* and one *bHLH* transcription factors were co-expressed with the expression of different genes. These genes and transcription factors may be responsible for the spatial color difference of *P. micranthum*. Our study emphasizes that the color of this food-deceptive orchids is achieved through specific genes and transcription factors associated with the pigment biosynthesis pathway.

## 1. Introduction

Flowering plants often rely on multi-sensory advertisements such as color, odor, and shape to attract pollinators and ensure successful reproduction. In turn, pollinators rely on these advertisements to locate flowers that offer rewards such as pollen, nectar, or oil [1]. However, some flowers are deceptive by mimicking the appearance of rewarding flowers without actually providing any rewards. Such flowers are considered as food deception [2].

The visual signals provided by deceptive flowers play a critical role in food deception [2,3,4,5]. The similarity in visual signals between rewarding flowers and deceptive species is a decisive factor in the pollination success of food deception [2,3,4,5]. Numerous studies have shown that olfactory signals can work together with visual signals to increase pollinator discrimination [5,6,7,8,9]. Conversely, several food-deceptive species that resemble the visual signals of model species produce scents that differ greatly to prevent inhibitory learning [10,11]. Therefore, the visual signal is the primary determinant of pollination success in food deception, while the role of olfactory signals varies among species [5,6,12].

Floral color is the most powerful visual signal in food deception [5,13,14,15]. The color signal, perceived by pollinators, is strongly determined by the type, concentration, and location of the pigment [16]. There are three major types of pigments—betalains, anthocyanins, and carotenoids—with betalains being restricted to the order of the Caryophyllales [17]. The other two pigments are widely found in angiosperms for the attractive natural display of flower colors and lay the groundwork for mimicking the color of rewarding species [18].

Anthocyanins and carotenoids can coexist within a single cell or in different regions of a flower [1,17]. Two biosynthesis pathways of pigments are conserved in angiosperms, with both genes in related biosynthesis pathways and transcription factors that regulate gene expression contributing to the concentration and location of pigments [19,20,21]. The accumulation of anthocyanins in vacuoles is strongly influenced by the expression level of any genes involved in the anthocyanin biosynthesis pathway (ABP) [20,22]. The transcription factor *R2R3-MYBs* forms a complex with *bHLH* and *WD40* and plays a crucial role in regulating gene expression in the ABP [23]. Carotenoids are primarily localized in the plastids of many flowers, with lutein and beta-carotene being the most commonly discovered pigments [24]. *R2R3-MYBs* have also been reported to play a role in regulating the expression of genes involved in the carotenoid biosynthesis pathway (CBP) [25,26,27].

Food deception strategy is frequently found in Orchidaceae [2,28]. About one-third of orchids employ food deception, indicating the efficacy of deceptive pollination strategy in orchids [29,30,31]. The visual signal is also the major factor in the pollination success of food deception orchids [5,6,12]. The pollination accomplishment of food deceptive orchids, *Anacamptis* (*Orchis*) *morio*, is influenced by the proximity to rewarding plants and the similarity of flower color to that of magnet plants, while also emitting a highly variable scent to avoid pollinator learning and selective behavior [10,11]. *Diuris brumalis* is a food-deceptive orchid that attracts pollinators using visual signals similar to *Daviesia decurrens* at close range and ultraviolet visual signals at long range [32,33].

*Paphiopedilum micranthum* (Cypripedioideae, Orchidaceae) is fascinating owing to its bright and striking floral displays, with bumblebees as their primary pollinators. Ma et al. demonstrated that the pollination success of *P. micranthum* is facilitated by the color resemblance of their flowers to the model plant *Rosa roxburghii* [4]. However, the metabolic and molecular bases underlying flower color in this species are poorly understood. In this study, we conducted targeted metabolic profiling and transcriptome analysis intended to reveal the formation and genetic basis of floral color in *P. micranthum*.

## 2. Results

### 2.1. Morphology and Phenotypic Modifications of P. micranthum during Floral Development

**Figure 1:**
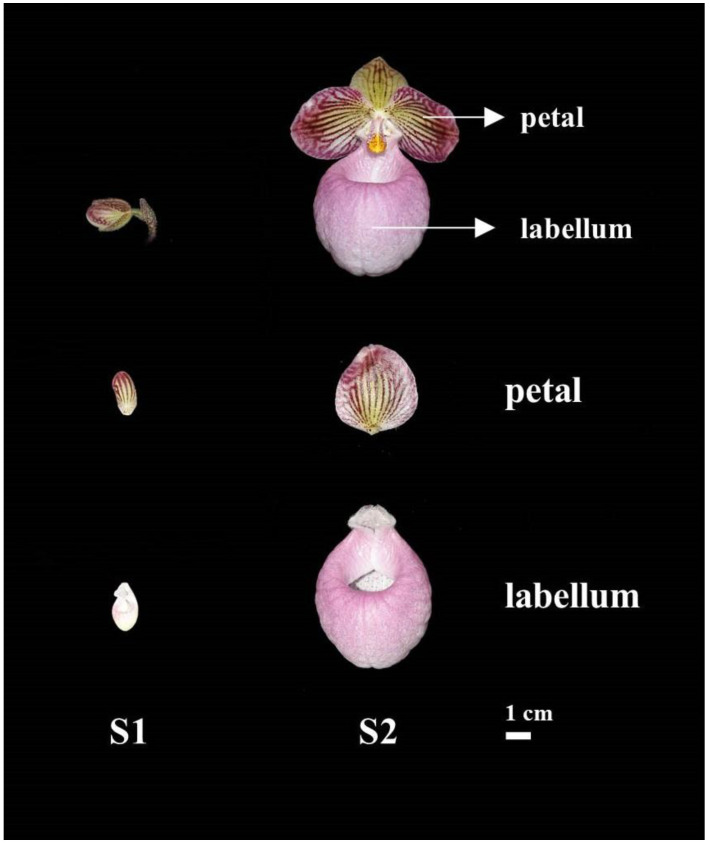
Phenotypic changes of *P. micranthum* flowers during floral development. S1, bud length 2–2.5 cm; S2, full-bloom stage.

The mature flowers of *P. micranthum* have two notable structures with distinct colors, a pink labellum and two purple petals with a yellow base (Figure 1). For the petals in stage 1 (i.e., bud length 2–2.5 cm, hereafter S1), dark purple and yellow coloration have already formed which turn into purple and bright yellow in stage 2 (i.e., full-bloom stage, hereafter S2). Meanwhile, the labellum changes from a pale pink to pink from S1 to S2.

### 2.2. Identification and Quantification of Pigment Components

**Figure 2:**
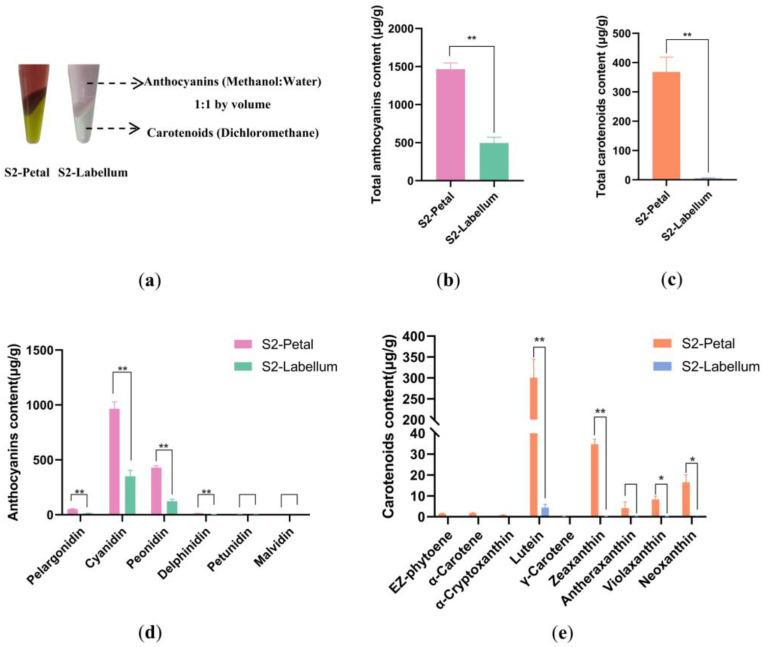
Targeted metabolome analyses of anthocyanins and carotenoids in the petal and labellum in S2. (**a**) The primary identification of pigments, (**b**) Different total anthocyanin content in the petal and labellum, (**c**) Different total carotenoid content in the petal and labellum, (**d**) Anthocyanin-targeted metabolome identified differentially accumulating compounds, (**e**) Carotenoid-targeted metabolome identified compounds that accumulate differently. “*” indicates *p* < 0.05, and “**” indicates *p* < 0.01.

To identify the primary pigments contributing to the coloration of petal and labellum in S2, pigment separation experiments were conducted (Figure 2a). Petals contain both anthocyanin and carotenoid responsible for the purple and yellow color, respectively. Anthocyanin was the primary pigment responsible for the pink coloration of the labellum with little carotenoid.

The targeted assay showed a considerable increase in total anthocyanidin of the petal compared to the labellum (Figure 2b). There were six types of anthocyanidin both in the petal and labellum, with significant amounts of cyanidin and peonidin (Figure 2d). The content of cyanidin was the highest both in the petal and labellum, followed by peonidin and pelargonidin. These findings revealed that a higher anthocyanin concentration in the petal contributed to dark purple coloration whereas a lower anthocyanin concentration contributed to pink coloration.

According to the targeted assay, the total carotenoid content in the petal significantly increased compared to the labellum (Figure 2c). The concentrations of lutein and zeaxanthin were the major compounds in the petal that contributed to the yellow coloration of the petal (Figure 2e).

Our findings suggested that the different coloration of *P. micranthum* was a result of the main accumulation of cyanidin via the ABP, as well as lutein and zeaxanthin produced by the CBP. To investigate the genetic changes in floral coloration, we collected petals and labellums in S1 and S2 for transcriptomics analysis.

### 2.3. Differentially Expressed Genes Related to the Pigment Biosynthesis

**Figure 3:**
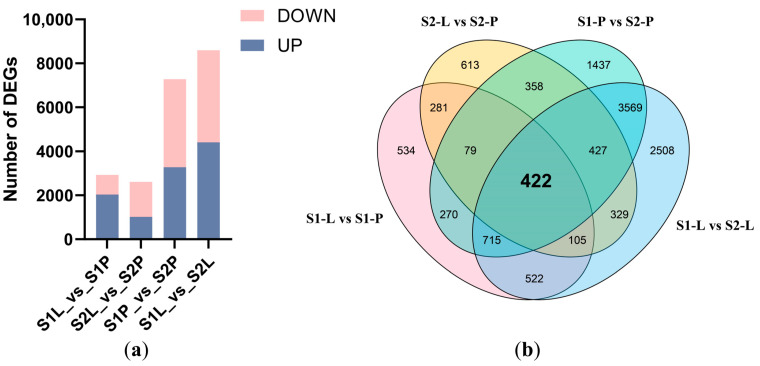
Transcriptomic features for *P. micranthum*. (**a**) The number of genes that are up- and down-regulated in four comparisons, (**b**) The Venn diagram illustrates the specific and shared numbers of DEGs present in the labellum and petal tissues. S1-P, the petal of S1, S1-L, the labellum of S1, S2-P, the petal of S2, S2-L, the labellum of S2.

Pairwise sample comparisons of transcriptomes from various floral parts in two developmental stages were conducted to identify differentially expressed genes (DEGs). We identified 2928, 2614, 7277, and 8597 DEGs in S1-L vs. S1-P, S2-L vs. S2-P, S1-P vs. S2-P, S1-L vs. S2-L groups, respectively (Figure 3a), while 422 DEGs were common in all comparisons (Figure 3b).

**Figure 4:**
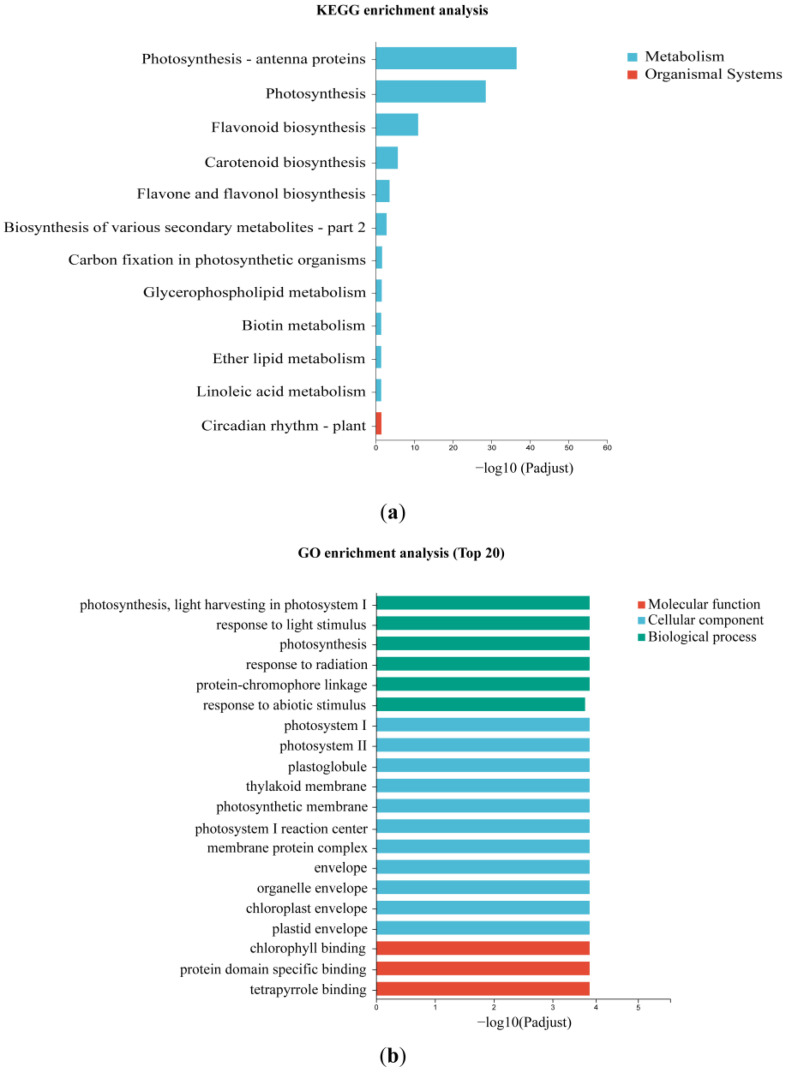
KEGG and GO enrichment analysis of common DEGs. (**a**) KEGG enrichment analysis. (**b**) GO enrichment analysis.

By using the Kyoto Encyclopedia of Genes and Genomes (KEGG) enrichment analysis, the 422 DEGs were grouped into 11 differential metabolic pathways and 1 organismal system. Photosynthesis-antenna proteins, photosynthesis, flavonoid, and carotenoid biosynthesis were the most distinct metabolic pathways (Figure 4a). The known results well supported the alternation of flavonoid biosynthesis, and carotenoid biosynthesis was the underlying factor leading to differential pigment accumulations between the petal and labellum during the floral development of *P. micranthum*. To acquire the functions of DEGs, the top 20 terms were shown through the Gene Ontology (GO) enrichment analysis, including 6 molecular function terms, 11 cellular component terms, and 3 biological process terms (Figure 4b). Most terms were related to photosynthesis.

### 2.4. DEGs Involved in the ABP and CBP

**Figure 5:**
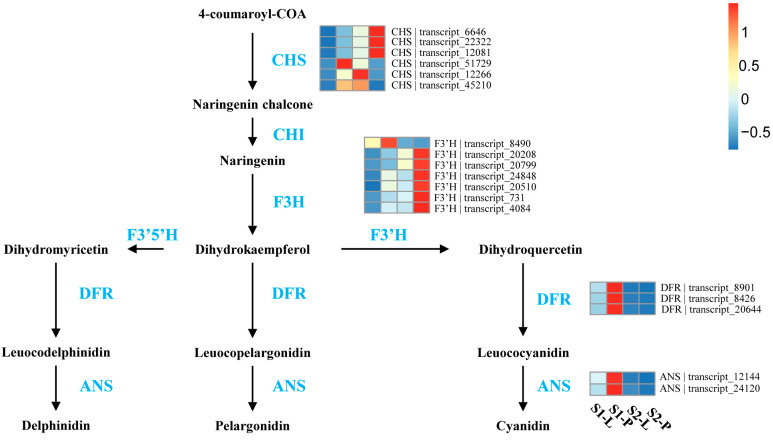
Overview of the anthocyanin biosynthesis pathway and the roles of DEGs in the flower of *P. micranthum*. The data are the mean TPM value of three biological replicates. *CHS*, chalcone synthase; *CHI*, chalcone isomerase; *F3H*, flavone 3-hydroxylase; *F3*′*H*, flavone 3′-hydroxylase; *F3*′*5*′*H*, flavone 3′5′-hydroxylase; *DFR*, dihydroflavonol reductase; *ANS*, anthocyanidin synthase. S1-P, the petal of S1, S1-L, the labellum of S1, S2-P, the petal of S2, S2-L, the labellum of S2. In scale bar, the expression of DEGs is indicated in red (high abundance) and blue (low abundance).

The expression heatmap of DEGs involved in anthocyanin pathways can be found in Figure 5. In S1, the expressions of *CHS*, *F3*′*H*, *DFR*, and *ANS* were up-regulated in the petal compared to the labellum, consistent with the color difference in the petals (purple) and labellum (pale pink). In S2, *CHS* and *F3*′*H* were further up-regulated in the petal compared to those in the labellum, consistent with darker coloration and higher anthocyanins in the petal than those in the labellum.

**Figure 6:**
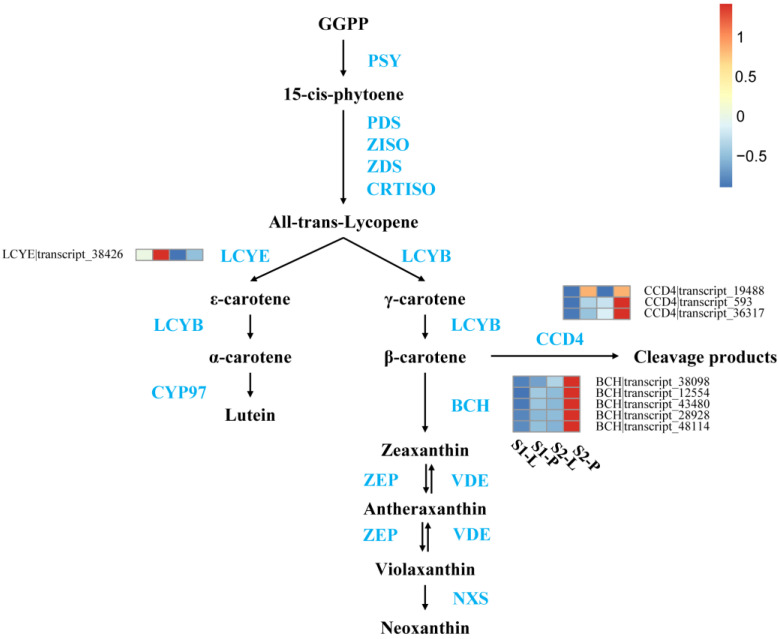
Overview of the carotenoid biosynthesis pathway and the roles of DEGs in the flower of *P. micranthum*. The data are the mean TPM value of three biological replicates. *PSY*, Phytoene synthase; *PDS*, phytoene desaturase; Z*-ISO*, zeta-carotene isomerase; *ZDS*, zeta-carotene desaturase; *LCYB*, lycopene beta-cyclase; *LCYE*, lycopene epsilon-cyclase; *CYP97*, cytochrome P450–type hydroxylase; *BCH*, beta-carotene hydroxylase; *ZEP*, zeaxanthin epoxidase; *VDE*, violaxanthin de-epoxidase; *NXS*, neoxanthin synthase; *CCD4*, carotenoid cleavage dioxygenase 4. S1-P, the petal of S1, S1-L, the labellum of S1, S2-P, the petal of S2, S2-L, the labellum of S2. In scale bar, the expression of DEGs is indicated in red (high abundance) and blue (low abundance).

In the metabolic analyses, lutein, zeaxanthin, violaxanthin, and neoxanthin were the main pigments that contributed to the yellow coloration of the petal, but *β*-carotene was not detected in the petal or labellum (Figure 2e). Correspondingly, the expression of *CCD4* and *BCH*, which causes *β*-carotene to be cleaved and zeaxanthin to be synthesized, respectively, was upregulated in the petal in S2 (Figure 6). In addition, the expressions of the DEGs of *LCYE*, *CCD4*, and *BCH* were upregulated in the petal both in S1 and S2, and were consistent with high carotenoid concentrations in the petal, resulting in a yellow color base.

### 2.5. Weighted Gene Coexpression Network Analysis (WGCNA)

**Figure 7:**
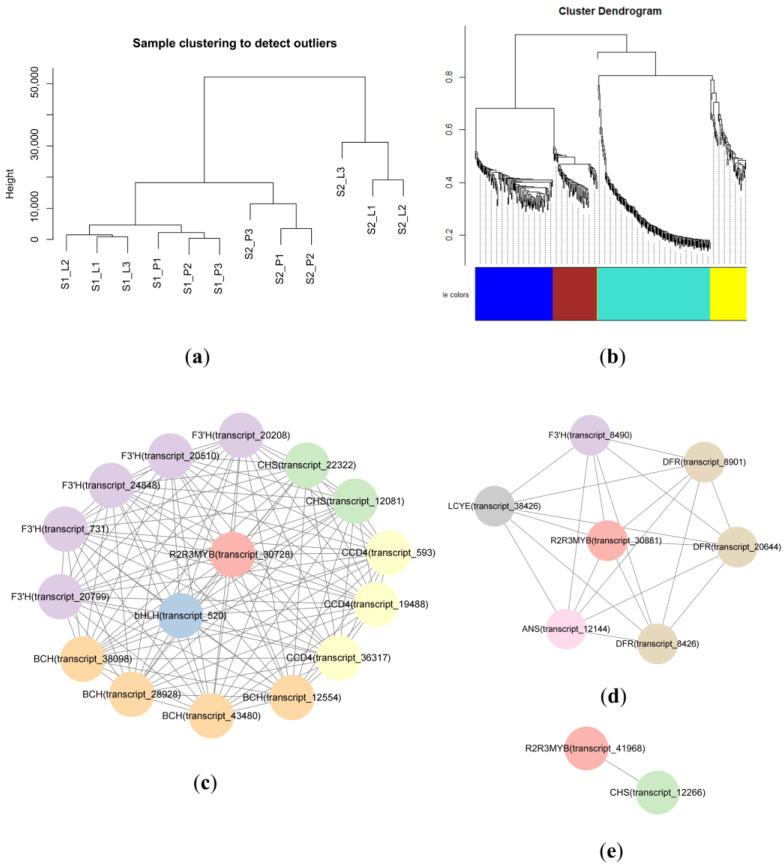
Module identification by using WGCNA. (**a**) Sample clustering analysis. (**b**) Gene dendrogram obtained by clustering the dissimilarity, based on consensus topological overlap with the corresponding module colors, indicated by the color row, and each colored row represents a color-coded module that contains a group of highly connected genes. (**c**) The blue module. (**d**) The turquoise module. (**e**) The yellow module.

The sample clustering analysis showed that all the samples were well clustered and no outliers were found (Figure 7a). A WGCNA of 422 DEGs showed contrasting modules regarding color regulation. The different expressions of structural genes and transcription factors related to ABP and CBP were discovered in the blue, turquoise, and yellow modules, but none were found in the brown module (Figure 7b). In the blue module, *R2R3-MYB* (transcript_30728) and *bHLH* (transcript_520) were co-expressed with these genes (*CHS*, *F3*′*H*, *BCH*, *CCD4*) (Figure 7c). In the turquoise module, *R2R3-MYB* (transcript_30881) presented a co-expression relationship with structural genes (*F3*′*H*, *DFR*, *ANS*, *LCYE*) (Figure 7d). *R2R3-MYB* (transcript_41968) was also co-expressed with *CHS* in the yellow module (Figure 7e). Three *R2R3-MYBs* (transcript_30728, transcript_30881, and transcript_41968) and one *bHLH* (transcript_520) might promote the expression of ABP and CBP structural genes.

## 3. Discussion

### 3.1. Different Concentration of Accumulated Pigments Instigate Phenotypic Modifications in P. micranthum

Purple hue in orchids is caused by anthocyanin, and the most frequent anthocyanin in them is cyanidin [34,35]. Anthocyanins contributed to the pink-to-purple color of the petal and labellum in *P. micranthum*, especially cyanidin. Additionally, the color hue is related to the pigment concentration; the change in the color of *Chrysanthemum morifolium* is correlated with the cyanidin concentration, with the more cyanidins being present, the darker is the color of the flower [36]. The various concentration of cyanidins might contribute to the dark purple coloration in the petal and the pink coloration in the labellum.

Distinct carotenoid species produce diversity in floral color, indicating that a lack of carotenoids causes white flowers, an accumulation of lutein and zeaxanthin causes yellow flowers, and the presence of *β*-carotene causes orange-red flowers [37,38]. The higher levels of lutein and zeaxanthin than those in the labellum, with little *β*-carotene in the petal and labellum, were consistent with the yellow coloration of the petal of *P. micranthum*.

### 3.2. Different Accumulation of Pigments Related to the Expression of the Structural Genes

By utilizing KEGG and GO enrichment analysis, DEGs were found to be significantly connected to photosynthesis. This difference could be attributed to the presence of chloroplasts in the floral tissues, which act as an energy source similar to those found in Phalaenopsis orchids [39]. However, most of all, the enrichment analysis found that DEGs were related to flavonoid and carotenoid biosynthesis pathways, suggesting that the differential expression of these genes may underlie the pigment accumulation between the petal and labellum during floral development.

The expression levels of structural genes in pigment biosynthesis pathways can have a direct impact on the amount of pigment accumulation, ultimately determining the color of the flower [20,25]. The results of the morphological and anatomical analysis indicate that the changes in the petal and labellum are different from each other (Figure 2). The petals initially exhibited a dark purple hue in S1 and gradually lightened in color as they matured. In S1, the expression of late biosynthetic genes, *DFR* and *ANS*, was strongly activated, whereas their activity was comparatively lower in S2. The difference was that as it developed, the color of the labellum became gradually darker. Furthermore, the different expression of genes *CHS* and *F3*′*H* in S1 and S2 might also affect the pigment accumulation.

In addition, the metabolomics analysis revealed a significantly higher accumulation of anthocyanins in the petals compared to that in the labellum. The reason for this difference in anthocyanin accumulation might be attributed to the higher expression levels of *CHS*, *F3*′*H*, *DFR*, and *ANS* in the petals in S1, and *CHS*, *F3*′*H*, and *DFR* in S2, than those in the labellum.

The expression of *LCYE* affects the accumulation of lutein, responsible for yellow coloration [24,40]. Based on our transcriptome analysis, *LCYE* expression levels were higher in the petal than in the labellum both in S1 and S2, indicating its correlation with increased lutein accumulation in the petal.

Furthermore, the upregulation of *BCH* in the petal compared to the labellum may result in higher concentrations of zeaxanthin, violaxanthin, and neoxanthin. Similar observations have been reported in *Dendrobium chrysotoxum* and *Ipomoea nil* [41,42]. Another upregulated gene in the petal is *CCD4*, which is accountable for the cleavage of *β*-carotene [37]. The increased expression of *CCD4* and *BCH* conceivably led to a reduction in the accumulation of *β*-carotene, thus preventing the petal from appearing with an orange-red hue. In *Eustoma grandiflorum*, the *CCD4* gene is highly expressed in both yellow and white petals. However, the *PSY* and *LCYB* genes are expressed at higher levels in yellow petals [43]. Therefore, we suggest that maintaining a balance between carotenoid production and degradation activities regulates the overall level of carotenoid accumulation in *P. micranthum*.

### 3.3. R2R3-MYBs and bHLH Regulate the Structural Genes of ABP and CBP

*R2R3-MYB* control pigment concentration and localization by regulating pigment biosynthesis genes [20,44]. In tree peony, *R2R3-MYB* interacts with *bHLH* and a *WD40* protein activates CHS expression, which causes purple spots to appear on white petals [45]. Moreover, the R2R3-MYB transcription factor, *RCP1*, regulates carotenoid synthesis in flowers [27]. In *P. micranthum*, we observed differential expression of three *R2R3-MYBs* and one *bHLH* transcription factors between the petal and labellum, and these transcriptome factors exhibited co-expression with structural genes involved in ABP and CBP, possibly responsible for the color pattern.

## 4. Materials and Methods

### 4.1. Plant Materials and Sampling

All flowers of *P. micranthum* used in this study were offered by Qianxinan Green Animal and Plant Science and Technology Development Company (Guizhou, China). We collected petals and labellums of *P. micranthum* in S1 and S2 (Figure 1) for metabolomics and transcriptome sequencing. We also collected flowers at different stages (i.e., bud length <1.5 cm, 1.5–2 cm, 2–2.5 cm, 2.5–3 cm, 3–3.5 cm, 3.5–4 cm, 4–4.5 cm, and full-bloom stage) for subsequent PacBio Iso-Seq. All samples were collected and immediately frozen in liquid nitrogen, and then stored at −80 °C.

### 4.2. Pigment Separation

To find out the main categories of floral pigments, 50 mg of the petal or labellum was ground into a powder in liquid nitrogen, dissolved into 200 µL methanol, and then mixed with 200 µL water and 200 µL dichloromethane [46]. Lastly, anthocyanins and carotenoids were separated, respectively, in the upper and lower layers after 5 min of centrifugation at 13,000 rpm.

### 4.3. Targeted Metabolome Profiling and Analysis

Anthocyanin contents were analyzed using MetWare (http://www.metware.cn/ (accessed on 21 May 2023)) based on the AB Sciex QTRAP 6500 LC-MS/MS platform. Three biological replicates were assessed. The freeze-dried samples were crushed to powder for 1.5 min at 30 Hz. A total of 0.5 mL of methanol/water/hydrochloric acid (500:500:1, *v*/*v*/*v*) was used to extract 50 mg of powder. The extraction was vortexed for 5 min, ultrasound for 5 min, and centrifuged at 12,000× *g* under 4 °C for 3 min. After filtration, the supernatants were used for the examination by using the UPLC-ESI-MS/MS system (UPLC, ExionLC™ AD, https://sciex.com.cn/ (accessed on 21 May 2023); MS, Applied Biosystems 6500 Triple Quadrupole, https://sciex.com.cn/(accessed on 21 May 2023)).

Carotenoid contents were detected using MetWare (http://www.metware.cn/ (accessed on 21 May 2023)) based on the AB Sciex QTRAP 6500 LC-MS/MS platform. Three biological replicates were assessed. The freeze-dried sample of carotenoid extraction was ground to powder for 1.5 min at 30 Hz. A total of 50 mg powder was extracted with 0.5 mL mixed solution of n-hexane: acetone: ethanol (1:1:1, *v*/*v*/*v*). After 20 min vortexing, the extract was centrifuged at 12,000 r/min for 5 min at 4 °C, and then the supernatants were collected. The supernatants were evaporated till they were dry and then, they were reconstituted in a mixed solution of MeOH/methyl tert-butyl ether (1:1, *v*/*v*). After filtration, the solution was used for further LC-MS/MS analysis.

For statistical analysis, the Student’s *t*-tests were performed using R to evaluate the differences in anthocyanin and carotenoid in the S2-P and S2-L, following the confirmation of normality using a Shapiro-Wilk test.

### 4.4. RNA Extraction, Library Preparation, Sequencing, and Annotation

Total RNA was extracted using TRIzol^®^ Reagent and DNA was removed using DNase I (TaKara, Beijing, China). We used 1% agarose gel electrophoresis to assess RNA degradation and contamination. The integrity and purity of the RNA samples were determined with the 2100 Bioanalyser (Agilent Technologies, Wilmington, DE, USA) and ND-2000 (NanoDrop Technologies, Wilmington, DE, USA), respectively.

PacBio Iso-Seq and Illumina sequencing were performed at the Shanghai Majorbio Bio-Pharm Technology Co (Shanghai, China). To obtain the reference transcripts, the total RNA of flowers at different stages was extracted, and the RNA of each sample was equally mixed. The mixed RNA reversed transcription to the full-length 1st strand cDNA using Clontech SMARTer PCR cDNA synthesis kit. After PCR amplification, DNA damage repair, terminal repair, adapter connection, and purification, products were sequenced on the PacBio Sequel platform. The circular consensus sequence (CCS) reads were extracted following the filtering of raw data. Then, to categorize the CCS and identify the full-length (FLNC) and non-full-length (nFL) nonchimeric sequences, we determined if the CCS comprised the 5′-primer, 3′-primer, and poly-A sequences. The FLNC was through clustered, corrected, and polished. Reference transcripts were created using the polished consensus sequence. The reference transcripts were annotated against the nonredundant protein (Nr) database (http://www.ncbi.nlm.nih.gov (accessed on 21 May 2023)), KEGG database (http://www.genome.jp/kegg (accessed on 21 May 2023)), and GO database (http://www.geneontology.org (accessed on 21 May 2023)).

The Illumina library was prepared following TruSeq TM RNA sample preparation Kit from Illumina (San Diego, CA, USA) using 1 μg of total RNA to obtain messenger RNA. Secondly, double-stranded cDNA was synthesized using a SuperScript double-stranded cDNA synthesis kit (Invitrogen, San Diego, CA, USA) with random hexamer primers (Illumina). After selection and amplification, the paired-end RNA-seq sequencing library was sequenced with the Illumina NovaSeq 6000 sequencer. The raw paired-end reads were trimmed and quality controlled using fastp (https://github.com/OpenGene/fastp (accessed on 21 May 2023)) with default parameters to obtain clean data.

### 4.5. DEGs, Functional Enrichment Analysis, and WGCNA

The clean data were mapped to the reference transcripts and the gene expression levels were estimated via the transcripts per kilobase of exon model per million mapped reads (TPM) method using StringTie v1.3.4 (https://ccb.jhu.edu/software/stringtie/index.shtml?t=example (accessed on 21 May 2023)).

The differential expression analysis was based on the TPM value, which represents the mean of three biological replicates. DEGs were identified using the DESeq2 software based on |log2(foldchange)| ≥ 1 and *p*-value ≤ 0.05. A total of 422 DEGs were identified, and then, we performed KEGG and GO enrichment analysis. Significantly enriched terms were selected at q value ≤ 0.05. The structural genes of anthocyanin synthesis pathway and carotenoid synthesis pathway were screened from 422 genes, and the heatmap was illustrated using R v4.1.2.

To analyze the gene co-expression network, 422 DEGs were analyzed via the WGCNA v4.1.3 by using R. The TPM values were imported into the WGCNA package to establish co-expression modules and the power was 4. *R2R3-MYB* and transcription factors, which may be related to anthocyanin and carotenoids, are screened from each module [42]. The networks were visualized using Cytoscape v3.9.1.

## 5. Conclusions

The floral color of the food-deceptive orchid *P. micranthum* varies in the petal and labellum. Our studies show that both anthocyanins and carotenoids contribute to the formation of floral color. Different amounts of pigments are uncovered in two floral parts. Higher concentrations of anthocyanins (cyanidin and peonidin) and carotenoids (primarily lutein and zeaxanthin) were detected in the petal compared to the labellum. The upregulations of structural genes of *CHS*, *F3*′*H*, *DFR*, and *ANS* on the anthocyanin biosynthesis pathway in petals as well as three genes of *LCYE*, *BCH*, and *CCD4* on the carotenoid biosynthesis pathway were identified. Furthermore, three *R2R3-MYBs* and one *bHLH* transcription factors were co-expressed with the structural genes. These genes and transcription factors may be responsible for the spatial color difference of *P. micranthum*. Further studies such as qRT-PCR and transient overexpression can be used to verify the function of these genes. Moreover, a former study demonstrated that the contrast between the inner (such as the staminode and the base of the petal) and the outer (such as the tip of the petal, labellum, and sepal) colors of *P. micranthum* are important during the foraging of bumblebees [4]. Therefore, more studies on the color of staminode should be performed to further reveal the function and molecular basis of the inner color.

## Data Availability

The raw data have been deposited in NCBI under BioProject accession number PRJNA918555.
